# Exposure Characteristics and Cumulative Risk Assessment for Phthalates in Children Living near a Petrochemical Complex

**DOI:** 10.3390/toxics11010057

**Published:** 2023-01-06

**Authors:** Chih-Wen Wang, Po-Keng Cheng, Vinoth Kumar Ponnusamy, Hung-Che Chiang, Wan-Ting Chang, Po-Chin Huang

**Affiliations:** 1 Division of Hepatobiliary, Department of Internal Medicine, Kaohsiung Medical University Hospital, Kaohsiung Medical University, Kaohsiung 701, Taiwan; 2 Department of Internal Medicine, Kaohsiung Municipal Siaogang Hospital, Kaohsiung Medical University, Kaohsiung 701, Taiwan; 3 Research Center for Precision Environmental Medicine, Kaohsiung Medical University, Kaohsiung 701, Taiwan; 4 Department of Finance and Cooperative Management, National Taipei University, New Taipei City 237, Taiwan; 5 Department of Medicinal and Applied Chemistry, Kaohsiung Medical University, Kaohsiung 807, Taiwan; 6 Department of Medical Research, China Medical University Hospital, China Medical University, Taichung 404, Taiwan; 7 National Institute of Environmental Health Sciences, National Health Research Institutes, Miaoli 350, Taiwan

**Keywords:** daily intakes, hazard index, hazard quotient, phthalate metabolites, petrochemical complex

## Abstract

Background: School-aged children living near plastics–producing factories may have higher risk of exposure to phthalates released during the manufacturing processes. Objectives: We aimed to investigate the urinary concentrations of phthalate metabolites in school-aged children living near a petrochemical complex and estimate the cumulative risk of phthalate exposure. Methods: We used a well-established cohort (Taiwan Petrochemical Complex Cohort for Children, TPE3C) of school-aged children (6–13 years old) living near polyvinyl chloride (PVC) and vinyl chloride monomer (VCM) factories in central Taiwan from October 2013 to September 2014. A total of 257 children were included from five elementary schools: Syu-Cuo Branch (*n* = 58, school A, ~0.9 km), Feng-An (*n* = 40, school B, ~2.7 km), Ciao-Tou (*n* = 58, school C, ~5.5 km), Mai-Liao (*n* = 37, school D, ~6.9 km), and Lung-Feng (*n* = 57, school E, ~8.6 km). We analyzed 11 metabolites of seven phthalates (including di-2-ethylhexyl phthalate (DEHP) and di-n-butyl phthalate (DnBP)) in urine. Daily intakes (DIs) were compared with acceptable intake levels to calculate the hazard quotient (HQ) for individual phthalates, and the cumulative risk for each child was assessed using a hazard index (HI), which was the sum of the the individual HQs. Results: The geometric mean and proportion of participants with HIs exceeding one for hepatic (HI_hep_) and reproductive (HI_rep_) effects were 0.33 (13.2%) and 0.24 (7.8%), respectively. The major contributors to phthalate exposure risk were DEHP, di-iso-butyl phthalate (DiBP) and DnBP in all children. Moreover, we observed a U shaped distribution of DEHP exposure by school distance from the PVC and VCM factories (school A: 7.48 μg/kg/day and school E: 80.44 μg/kg/day). This may be due to emissions (closest) and and being located downwind of PVC scrap incineration (farthest). Conclusions: Our findings suggest that children living near a petrochemical complex were at a greater risk of phthalate exposure than normal school-aged children and that phthalate exposure was mainly attributed to DEHP, DiBP and DnBP. In addition, inhalation may have been a risk factor for people living near to PVC and VCM factories.

## 1. Introduction

Phthalates are polyvinyl chloride (PVC)-containing chemicals commonly used to increase the flexibility of plastics in various consumer products. High molecular weight phthalates, such as di-iso-nonyl phthalate (DiNP) and di-iso-decyl phthalate (DiDP) have been used in flooring and building materials, garden hoses, shoes, and toys. Butyl benzyl phthalate (BBzP) and di(2-ethylhexyl) phthalate (DEHP) have been banned in the manufacture of toys for children, but are still used to make vinyl-flooring products, food packing materials, and a variety of medical plastic devices. Low molecular weight phthalates, such as dimethyl phthalate (DMP), diethyl phthalate (DEP), and di-*n*-butyl phthalate (DnBP), are used in cosmetics, personal care products, perfumes, creams, candles, shampoos, and surface-coating materials [1].

The major route of exposure to most phthalates is food ingestion; however, other minor routes of phthalate exposure include inhalation, drinking contaminated water, and absorption through the skin [2,3]. People living near phthalate-producing factories or hazardous waste sites may be exposed to phthalates released into the air or ground water [4]. Studies have revealed that the deposition rate for DEHP decreases with increasing distance from a smokestack at a phthalate-consuming factory [4]. The estimated quantities of vinyl chloride monomer (VCM) and PVC produced at the No. 6 Naphtha Cracking Complex in central Taiwan were 2.76 and 2.93 million tons, respectively, and the estimated annual emissions of VCM and 1,2-dichloroethane from the stack and equipment were 24.9 and 11.5 tons, respectively.

Previous nationwide human biomonitoring studies have revealed that phthalate exposure levels among a 7–17-year-old group in Taiwan were higher than those among individuals in the United States (NHANES 2015–2016), Canada (CHMS 2016–2017), and Germany (GerES V 2014–2017), particularly for DMP, DBP, and DEHP metabolites [5]. Moreover, participants from central Taiwan had higher phthalate exposure levels than did those from other areas [5]. Research has revealed that multifactorial phthalate exposure in pregnant women is associated with temporal trends and geographic variation across countries [6], but phthalate exposure variations among children remain unclear. Previously, our group demonstrated that school-aged children living nearest to a petrochemical complex had the highest urinary thiodiglycolic acid levels [7] and an increased risk of liver fibrosis [8] and non-alcoholic fatty liver disease [9].

The hazard index (HI) is the sum of the hazard quotients (HQs) of individual chemicals in a mixture, and an HI exceed one is considered indicative of potential adverse health effects [10,11]. The HQ of a chemical is estimated as the ratio of the calculated exposure level to the reference exposure values (RfVs) for that chemical. A study revealed that an HI of >1 derived for plasticizers with antiandrogen-based reference doses was exhibited by 86%, 80%, and 49% of Saudi, Indonesian, and Thai children, respectively, where DEHP was identified as a common major risk factor for children in all three countries, followed by DnBP and DiBP [12]. Another study in China demonstrated that at least 36% of children from a manufacturing-intensive region had an HI higher than one [13].

The aim of this study was to (1) investigate urinary concentrations of phthalate metabolites (mPAEs) in children living near PVC and VCM factories and (2) estimate their cumulative risk of exposure to phthalates.

## 2. Materials and Methods

### 2.1. Ethics Statement

The study protocol was approved by the Institutional Review Board of the National Health Research Institutes (No. EC1020607). Prior to study enrollment, all children provided informed consent and the parents of the children signed an additional agreement.

### 2.2. Participants and Study Design

**Figure 1:**
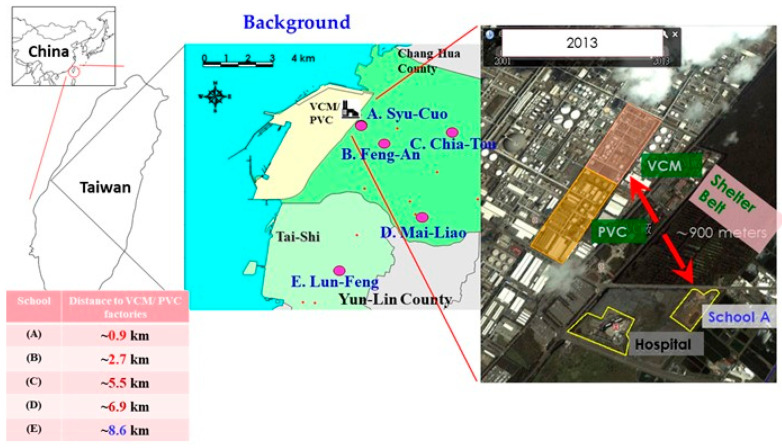
Sample elementary schools (A–E) near polyvinyl chloride (PVC) and vinyl chloride monomer (VCM) factories in central Taiwan.

We used a well-established cohort (Taiwan Petrochemical Complex Cohort for Children, TPE3C) of school-aged children (6–13 years old) living near VCM and PVC factories in a petrochemical complex in Yunlin County, central Taiwan, from October 2013 to September 2014 [7,8]. A total of 343 children were chosen from five elementary schools: Syu-Cuo Branch (*n* = 69, school A, ~0.9 km), Feng-An (*n* = 59, school B, ~2.7 km), Ciao-Tou (*n* = 67, school C, ~5.5 km), Mai-Liao (*n* = 75, school D, ~6.9 km), and Lung-Feng (*n* = 73, school E, ~8.6 km; Figure 1). Each student was then randomly matched by sex using their school identification number. The following individuals were enrolled: students in grades 1–6 of elementary school with a minimum of 1 year of local residence and a minimum age of 6 years. Children who had not fasted for at least 10 h (*n* = 39) were initially excluded. Then, in accordance with World Health Organization (WHO) guidelines, we excluded samples that exhibited creatinine concentrations below 30 mg/dL or above 300 mg/dL for valid urine samples (*n* = 27) [14]. Children who had consumed vitamin supplements less than 1 week before the study (*n* = 5) or who had chronic hepatitis B or C (*n* = 1) were excluded. We also excluded children with insufficient biochemistry data (*n* = 14). In total, 257 children were included in the final study.

### 2.3. Analytical Method

Urine samples for the phthalate metabolite analysis were collected in polypropylene containers and stored at −80 °C until analysis. Liquid chromatography–electrospray tandem mass spectrometry (LC-ESI-MS/ MS) [15] was employed to measure the concentrations of 11 phthalate metabolites, namely monoethylhexyl phthalate (MEHP), mono-(2-ethyl-5-oxo-hexyl) phthalate (MEOHP), mono-(2-ethyl-5-hydroxyhexyl) phthalate (MEHHP), mono-(2-ethyl-5-carboxypentyl) phthalate (MECPP), mono-(2-carboxymethylhexyl) phthalate (MCMHP), mono-n-butyl phthalate (MnBP), mono-iso-butyl phthalate (MiBP), mono-ethyl phthalate (MEP), monoiso-nonyl phthalate (MiNP), MBzP, and mono-methyl phthalate (MMP). A urine sample (100 μL) was incubated at 37 °C for 90 min with ammonium acetate (20 μL, >98%; Sigma-Aldrich, St. Louis, MO, USA), β-glucuronidase (10 μL, E. coli K12; Roche Biomedical, Germany), and a mixture of 10 isotopic (^13^C_4_) phthalate metabolite standards (100 μL, Cambridge Isotope Laboratories, Andover, MA, USA). LC-ESI-MS/MS (Agilent 1200/API 4000; Applied Biosystems, Foster City, CA, USA) was used together with an online system. The limits of detection (LOD) for MMP, MEP, MiBP, MnBP, MBzP, MEHP, MEHHP, MEOHP, MECPP, MCMHP, and MiNP were 0.3, 0.3, 1.0, 1.0, 0.3, 0.7, 0.3, 0.3, 0.3, 0.1, and 0.1 ng/mL, respectively. One blank repeated quality control (QC) sample and one spiked QC sample were included in each batch. The blank sample concentration was lower than twice the LOD. The QC sample for each sample batch in pooled urine samples was spiked with a mixture of phthalate metabolite standards (20–50 ng/mL). The relative percentage difference of the QC sample was less than ±30%, and the recovery rate of the QC sample was 100 ± 20%. Concentration values below the calibration curve were analyzed as 1/2 LOD values [16].

### 2.4. Estimation of Phthalates Daily Intake of 

We estimated the daily intake (DI) of each phthalate using urinary phthalate metabolites. The formula is represented by Equation (1) [17], where UE is the urinary excretion of the measured urinary phthalate metabolites per gram of creatinine; CE_smoothed_ is the smoothed creatinine excretion rate, which is an age, body weight (BW), height (ht), and sex-based value for urinary creatinine excretion rate used in [18,19]; F_UE_ is the molar fraction, which describes the molar ratio between the excreted amounts of the specific metabolites of each phthalate corresponding to the dietary intake of the parent phthalate; MW_d_ is the molar weight of the diester parent compounds; and MW_m_ is the molar weight of the corresponding monoesters.

(1) $$\mathrm{Daily}\;\mathrm{intake}\;\left(\mu g/kg/day\right)=\frac{UE\left(\mu g/g\;crea\right)\times CE_{\mathrm{smoothed}}\left(mg/day\right)}{F_{UE}\times BW\left(kg\right)\times 1000\left(mg/g\right)}\times \frac{MW_{d}}{MW_{m}}$$

$$\begin{array}{l} \mathrm{For}\;\mathrm{minors}\;(\geq 6-<18\;\mathrm{years}\;\mathrm{old}):\\ CE_{\mathrm{smoothed}}=ht\times \left\{6.265+0.0564\times \left(ht-168\right)\right\}\ldots ht<168\;cm\ldots \left(\mathrm{male}\right)\\ CE_{\mathrm{smoothed}}=ht\times \left\{6.265+0.2550\times \left(ht-168\right)\right\}\ldots ht\geq 168\;cm\ldots \left(\mathrm{male}\right)\\ CE_{\mathrm{smoothed}}=2.045\times ht\times \exp\left\{0.01552\times \left(ht-90\right)\right\}\ldots \left(\mathrm{female}\right) \end{array}$$

For DEHP DI, the formula is represented by Equation (2), where UE is the urinary excretion of the measured total urinary DEHP metabolites per gram of creatinine [20].

(2) $$\mathrm{Daily}\;\mathrm{intake}\;\left(\mu g/kg/day\right)=\frac{UE\left(moles/g\;crea\right)\times CE_{\mathrm{smoothed}}\left(mg/day\right)\times MW_{d}}{F_{UE}\times BW\left(kg\right)\times 1000\left(mg/g\right)}$$

### 2.5. Hazard Quotients and Hazard Index of Phthalates

We utilized HQs to calculate each participant’s risk of exposure to each phthalate. The formula for HQs is as follows [12].

(3) $$HQ=\frac{DI}{\mathrm{Reference}\;\mathrm{limit}\;\mathrm{value}}$$

An HI lower than 1indicates a low probability of adverse effects from exposure to several chemicals [21]. The HI for cumulative hepatic effect derived from the reference doses (RfDs) is the sum of the HQs of DEHP, DiNP, and BBzP. The HI for cumulative reproductive effect derived from the tolerable daily intake (TDI) is the sum of the HQs of DEHP, DnBP, DiBP, and BBzP [12].

(4) $$HI_{hep}=HQ_{DEHP}+HQ_{DiNP}+HQ_{BBzP}$$

(5) $$HI_{rep}=HQ_{DEHP}+HQ_{DnBP}+HQ_{DiBP}+HQ_{BBzP}$$

### 2.6. Statistical Analysis

We used the Kruskal–Wallis test to examine the differences in continuous variables for all participants at the five elementary schools. The Chi-squared test was employed to assess the difference in categorical variables for all participants at the five elementary schools. We compared differences in participant urinary phthalate metabolite levels between the five elementary schools using the Kruskal–Wallis test. We also applied ANCOVA (adjusting for confounders) to compare the differences in urinary phthalate metabolite levels, DIs of phthalates, HQs of phthalates, and the HI for phthalates of our participants. R version 4.1.0 (R Foundation for Statistical Computing, Vienna, Austria) was used to conduct all statistical analyses.

## 3. Results

### 3.1. Demographic Characteristics of Participants

Table 1 shows the demographic characteristics of the participating students in this study. We enrolled 6.1–12.5-year-old elementary school students with a mean age of 10.1 years. The participants’ sex ratio was approximately even (boys,52.2%; girls,47.8%), and they had a mean body mass index (BMI) of 18.1 with a range of 12.2 to 32.7. Most of the parents of the participants had completed senior high school (~46%), ~37% had completed junior high school, and ~16% had a university degree. Approximately half of the parents had an annual family income of less than USD 15,600, and more than 20% of the parents had worked at the petrochemical complex. Approximately 65% of the children were exposed to passive smoke, and nearly 60% of the children self-reported having been exposed to an unknown odor in their neighborhood.

### 3.2. Distributions of Urinary Phthalates

Table 2 presents the levels of phthalate metabolites measured in this study. The detection rate of the phthalate metabolites was lowest for MiNP (school B, 7.5%) and highest for MEHHP (school B, 100%). The geometric means (GMs;ng/mL) of the phthalate metabolite concentrations among school-aged children were 14.12, 10.23, 10.95, 17.26, 0.75, 16.05, 26.19, 10.47, 33.15, 10.63, 0.77, 0.51 (nmole/mL) and 0.14 (nmole/mL) for MMP, MEP, MiBP, MnBP, MBzP, MEHP, MEHHP, MEOHP, MECPP, MCMHP, MiNP, ΣDEHPm and ΣDBPm, respectively. We found that children at school A exhibited the highest concentrations of MiBP, MBzP, MEOHP, MiNP, and ΣDBPm (MiBP: 16.33 ng/mL, *p* = 0.003; MBzP: 3.79 ng/mL, *p* < 0.001; MEOHP: 22.30 ng/mL, *p* < 0.001; MiNP: 8.67 ng/mL, *p* < 0.001; ΣDBPm: 0.21 nmole/mL, *p* < 0.001); children at school D exhibited the highest concentrations of MMP, MnBP, and MCMHP (MMP: 28.59 ng/mL, *p* < 0.001; MnBP: 25.47 ng/mL, *p* = 0.003; MCMHP: 22.89 ng/mL, *p* < 0.001),and children at school E exhibited the highest concentrations of MEHP, MECPP, and ΣDEHPm (MEHP: 208.92 ng/mL, *p* < 0.001; MECPP: 80.77 ng/mL, *p* < 0.001; ΣDEHPm: 1.22 nmole/mL, *p* < 0.001).

### 3.3. Distributions of Estimated DIs, HQs, and HI of Phthalates

**Figure 2:**
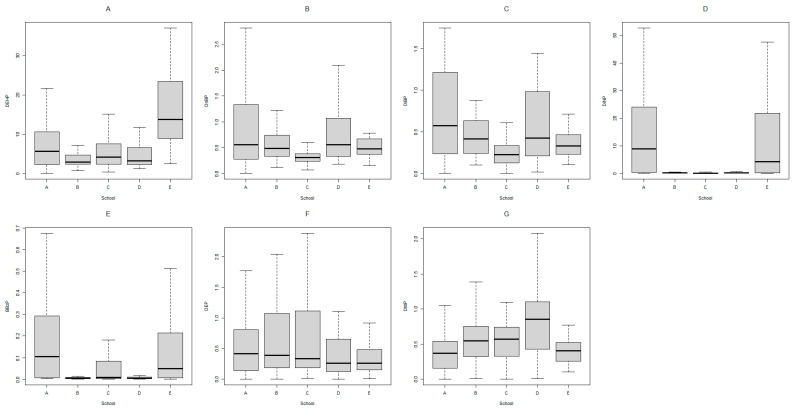
Estimated daily intakes (μg/kg/day) for seven PAEs (**A**–**G**) of participants by elementary school group (*n* = 257).

Figure 2 and Table S1 show the estimated DIs of phthalates, and Table 3 shows the distributions of HQs and the HI of phthalates by TDI. The GM DI levels of school-aged children were 5.60, 0.47, 0.36, 0.95, 0.02, 0.32 and 0.40 for DEHP, DnBP, DiBP, DiNP, BBzP, DEP and DMP, respectively. For children from specific schools, DIs (median, μg/kg/day) of DEHP (school E, 13.75; *p* < 0.001), DnBP (school D, 0.56; *p* < 0.001), DiBP (school A, 0.57; *p* < 0.001), DiNP (school A, 8.94; *p* < 0.001), BBzP (school A, 0.10; *p* < 0.001) and DMP (school D, 0.86; *p* < 0.001) were significantly higher than those of children from other schools. The GM HQ levels of school-aged children were 0.11, 0.05, 0.04, 0.02, 0.0004 and 0.0006 for DEHP, DnBP, DiBP, DiNP, BBzP and DEP, respectively. The GM of the HI_hep_ was 0.33, and 13.2% of participants had an HI_hep_ greater than 1; the GM of the HI_rep_ was 0.24, and 7.8% of participants had an HI_rep_ greater than 1.

### 3.4. Comparison of Phthalates Concentrations, DIs, HQs, and HI of Children between Schools

Using ANCOVA, after adjustment for urinary TDGA, urinary creatinine, age, sex, passive smoking exposure, BMI, parental employment at the petrochemical complex, and home location close to a main road, we found that the phthalate metabolite concentrations (mean, ng/mL) of MEHP (school E, 1384.67; *p* = 0.004) and ΣDEHPm (school E, 5.64 nmole/mL, *p* = 0.004) were significantly higher among children at school E than among children at other schools (Table S2). This trend was consistent for phthalate metabolite concentrations among all groups (Tables S3 and S4). The DIs (mean, μg/kg/day) of DEHP (school E, 80.44; *p* = 0.011) and DMP (school D, 1.28; *p* = 0.013) of children at schools E and D, respectively, were significantly higher than those of children from other schools after adjustment for confounders (Table 4). Moreover, we observed a U shaped distribution of the DEHP exposure by school distance from the PVC and VCM factories (school A: 7.48 μg/kg/day and school E: 80.44 μg/kg/day). This trend was consistent for DIs among all groups (Table S5). The HI_hep_ (median) and HI_rep_ (median) values for children at school E (HI_hep_: 4.22; *p* = 0.010; HI_rep_: 1.77; *p* = 0.014) were significantly higher than those for children at other schools (Table 4). We also observed a U shaped distribution of HI_hep_ exposure by school distance from the PVC and VCM factories (school A: 0.53 and school E: 4.22). This trend was consistent for HI_hep_ (median) and HI_rep_ (median) values among all groups (Table S6).

## 4. Discussion

This is the first study to assess the risk of exposure to phthalates in children living near a petrochemical complex. We measured the phthalate DIs, HQs and HIs for school-aged children from five elementary schools in the vicinity of PVC and VCM factories in central Taiwan. The children at school E (farthest from PVC and VCM factories) and school A (closest to PVC and VCM factories) had a significantly higher risk of HIs exceeding one.

Concentrations of the airborne phthalate DEHP are influenced by temperature in the vapor phase, are also associated with particulate mass concentrations, and are subject to both wet (rain or snow) and dry (wind or settling) deposition on the Earth’s surface [4,22]. This dispersion is likely due to particle-sorbed DEHP not reacting rapidly with hydroxyl radicals; however, vapor-phase DEHP reacts rapidly with hydroxyl radicals in the atmosphere [23]. The annual monitoring data from Taiwan’s EPA for air pollution in the area surrounding the petrochemical complex were examined. The annual mean level of VCM in the ambient air was 2.2 ppb (maximum level, 165 ppb) at school A, whereas that of 1-1dichloroethane was approximately one-fifth of the VCM level. Air monitoring stations near the other schools (B, C, D, and E) revealed a similar phenomenon (Table S7 and Figure S1). A study in central Taiwan demonstrated that spatial variations in particle-phase polycyclic aromatic hydrocarbon (PAH) concentrations occurred in the vicinity of the petrochemical complex occurred during seasonal downwind weather patterns [24]. The study produced consistent findings from air and biological monitoring, suggesting that the petrochemical complex was a major source of PAH exposure for the area and residents in its vicinity and that the increased external PAH levels in air might have contributed to elevated urinary 1-OHP levels in residents living near the complex. Another study further demonstrated that because of southerly winds blowing fumes from smokestacks of the complex, pollutants were carried northward to Tai-Si Village, the nearest village in Da-Cheng Township to the complex. This caused higher concentrations of V, Cr, Mn, Ni, Cu, As, Cd, and Tl and higher all-cause cancer incidence among Tai-Si residents [25]. Therefore, the highest levels of total phthalates, DEHP, and MEHP among school E children may have resulted from inhalation or dust ingestion. Thus, the spatial variation in phthalate metabolite concentrations in our study suggested inhalation as an additional exposure source. However, phthalate dispersion from the PVC and VCM factories and concentrations in the air near the petrochemical complex was unclear and require further investigation.

**Figure 3:**
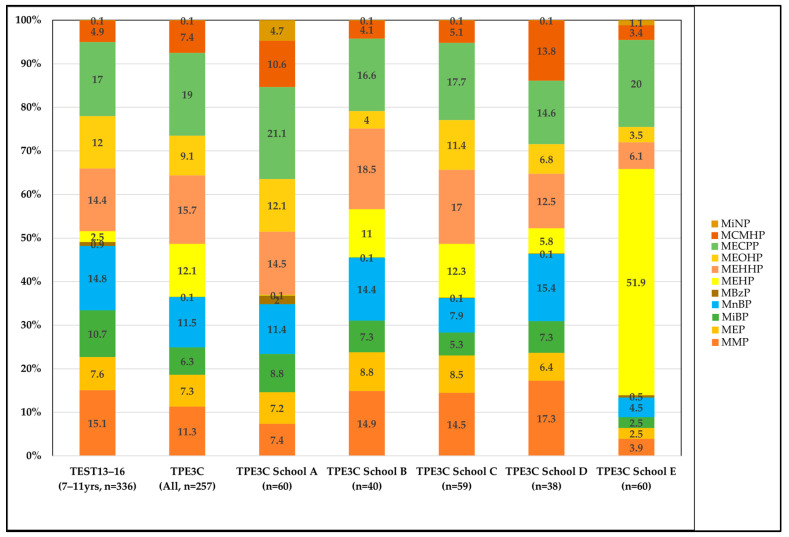
Comparison of the weights (%) of phthalate exposure in TEST13-16 7–11-year-olds and TPE3C participants.

Compared with that of children sampled from 22 cities and counties in Taiwan in a previous study [12], the proportion of participants living near to a petrochemical complex in this study with an HI exceeding one was higher (13.2% and 7.8% vs. 5.6%). However, the proportion of children in our study with an HI exceeding one was lower than that in other countries (86%, 80%, 49%, and 36% of Saudi, Indonesian, Thai and Chinese children, respectively). In addition, children living near to a region with intensive consumer goods manufacturing in Yuhuan, China, had HI values two to three times higher than those in the two other regions with results comparable to ours in a study [13]. Figure 3 shows compared weights (%) of phthalate exposure in 7–11-year-old individuals fromTEST13-16 (Liao et al., 2021) and TPE3C. Children at school E had a higher total phthalate exposure than that of TEST13-16 7–11-year-olds, and MEHP exposure at school E was considerably higher. These results suggest that individuals in manufacturing-intensive regions are likely to be at greater health risk from phthalate exposure and should be prioritized for intervention. Major risk contributors among the plasticizers in our study were DEHP, DiBP and DnBP, which is comparable to the studies of Saudi, Thai, Indonesian, Chinese and Brazilian children [13,26,27]. DEHP has frequently been identified as a major risk-driving phthalate in general populations [28] and pregnant women [29]. Therefore, identification of exposure sources of DEHP among children is the most critical factor in risk management for phthalate exposure.

The strength of this study is that we evaluated the risk of exposure to phthalates in school-aged children living near PVC and VCM factories. We assessed the spatial variation in phthalate exposure using human biomonitoring of our participants. This study has some limitations. First, we did not measure ambient air levels of the phthalates (this could be achieved by collecting outdoor and indoor PM_2.5_ samples). Second, the wind direction did not vary during our sampling period, autumn, and spring. Third, we did not utilize a detailed questionnaire regarding food contamination by phthalate, including exposure from containers and personal care products.

## 5. Conclusions

This is the first study to evaluate urinary phthalate metabolites in school-aged children living near PVC and VCM factories. Our findings suggest that children living near the petrochemical complex were at a greater health risk of phthalate exposure than were general school-aged children in Taiwan and that exposure to DnBP, DiBP, and DEHP constituted the majority of phthalate exposure. In addition, inhalation may have been a risk factor for individuals living near PVC and VCM factories. These findings should spur action to reduce the phthalate exposure risk in school-aged children, especially in PVC- and VCM- producing regions. However, whether inhalation or dust ingestion increases exposure to phthalates warrants further investigation.

## Figures and Tables

**Table 1:** Demographic characteristics of participants from five elementary schools (*n* = 257).

Characteristics/ Students from Schools	All (*n* = 257)	A (*n* = 60)	B (*n* = 40)	C (*n* = 59)	D (*n* = 38)	E (*n* = 60)	*p* ^a^
Continuous variables							
Age (y)	10.1 (6.1–12.5)	10.3 (6.5–12.1)	10.2 (6.3–12.1)	10.9 (6.9–12.5)	9.8 (6.9–12.4)	9.1 (6.1–12.0)	0.008 *
Body mass index (kg/m^2^)	18.1 (12.2–32.7)	16.9 (12.8–28.8)	18.4 (12.2–29.3)	19.0 (14.6–32.7)	17.5 (12.7–27.8)	18.6 (12.6–31.8)	0.149
Categorical variables [*n* (%)]							
Gender	.	.	.	.	.	.	0.440
Male	126 (49.0)	27 (45.0)	25 (62.5)	28 (47.5)	19 (50.0)	27 (45.0)	.
Female	131 (51.0)	33 (55.0)	15 (37.5)	31 (52.5)	19 (50.0)	33 (55.0)	.
Father’s education							0.232
≤Junior high school	98 (38.1)	26 (43.3)	14 (35.0)	20 (33.9)	15 (39.5)	23 (38.3)	.
Senior high school	118 (45.9)	28 (46.7)	19 (47.5)	32 (54.2)	12 (31.6)	27 (45.0)	.
≥University	40 (15.6)	5 (8.3)	7 (17.5)	7 (11.9)	11 (28.9)	10 (16.7)	.
Mother’s education							0.370
≤Junior high school	93 (36.2)	28 (46.7)	13 (32.5)	19 (32.2)	12 (31.6)	21 (35.0)	.
Senior high school	121 (47.1)	24 (40.0)	20 (50.0)	32 (54.2)	15 (39.5)	30 (50.0)	.
≥University	43 (16.7)	8 (13.3)	7 (17.5)	8 (13.6)	11 (28.9)	9 (15.0)	.
Annual family income (USD)							0.095
≤15,600	131 (51.0)	36 (60.0)	15 (37.5)	32 (54.2)	16 (42.1)	32 (53.3)	.
15,600–31,250	90 (35.0)	21 (35.0)	18 (45.0)	19 (32.2)	17 (44.7)	15 (25.0)	.
≥31,250	36 (14.0)	3 (5.0)	7 (17.5)	8 (13.6)	5 (13.2)	13 (21.7)	.
Father’s job ^b^	.	.	.	.	.	.	0.108
Yes	130 (50.6)	35 (58.3)	15 (37.5)	34 (57.6)	21 (55.3)	25 (41.7)	.
No	127 (49.4)	25 (41.7)	25 (62.5)	25 (42.4)	17 (44.7)	35 (58.3)	.
Mother’s job ^b^	.	.	.	.	.	.	0.469
Yes	53 (20.6)	16 (26.7)	9 (22.5)	13 (22.0)	7 (18.4)	8 (13.3)	.
No	204 (79.4)	44 (73.3)	31 (77.5)	46 (78.0)	31 (81.6)	52 (86.7)	.
Passive smoking	.	.	.	.	.	.	0.966
Yes	166 (64.6)	40 (66.7)	27 (67.5)	38 (64.4)	23 (60.5)	38 (63.3)	.
No	91 (35.4)	20 (33.3)	13 (32.5)	21 (35.6)	15 (39.5)	22 (36.7)	.
Unknown odor	.	.	.	.	.	.	0.119
Yes	155 (60.3)	42 (70.0)	28 (70.0)	30 (50.8)	23 (60.5)	32 (53.3)	.
No	102 (39.7)	18 (30.0)	12 (30.0)	29 (49.2)	15 (39.5)	28 (46.7)	.
Physical activity	.	.	.	.	.	.	<0.001 *
Low	95 (37.0)	33 (55.0)	24 (60.0)	13 (22.0)	7 (18.4)	18 (30.0)	.
Moderate	113 (44.0)	21 (35.0)	12 (30.0)	22 (37.3)	19 (50.0)	39 (65.0)	.
High	49 (19.0)	6 (10.0)	4 (10.0)	24 (40.7)	12 (31.6)	3 (5.0)	.

^a^ Kruskal–Wallis test for continuous variables and Chi-squared test for categorical variables, * *p* < 0.05. ^b^ We asked whether participants’ fathers or mothers had ever worked at the petrochemical complex.

**Table 2:** Distributions of phthalate (ng/mL) in participants by elementary school groups (*n* = 257).

mPAEs/	*n*	%>LOD ^a^	GM (95%CI)	Min	Selected Percentiles				Max	*p*-Value ^b^
Schools					25th (95%CI)	50th (95%CI)	75th (95%CI)	95th (95%CI)		
**MMP**										<0.001 ***
All	257	96.1	14.40 (12.28–16.88)	ND	9.49 (7.87–11.84)	17.82 (15.68–19.44)	27.81 (24.46–30.73)	84.97 (62.56–110.46)	408.02	
A	60	90.0	7.92 (5.17–12.12)	ND	5.95 (1.57–7.84)	13.73 (7.84–18.06)	20.39 (18.06–24.57)	47.90 (27.69–137.30)	137.30	
B	40	97.5	15.53 (11.37–21.20)	ND	11.61 (7.33–16.06)	19.52 (14.53–22.79)	25.66 (22.06–30.76)	36.74 (30.76–62.00)	62.00	
C	59	96.6	17.86 (12.88–24.77)	ND	11.41 (8.08–14.40)	21.27 (15.06–26.55)	32.04 (26.85–35.55)	105.85 (45.31–235.44)	235.44	
D	38	97.4	25.68 (16.21–40.66)	ND	13.84 (7.87–21.72)	28.59 (19.22–34.48)	49.37 (33.39–91.24)	181.00 (91.24–408.02)	408.02	
E	60	100	13.96 (11.55–16.88)	2.59	9.45 (6.99–12.77)	15.78 (12.77–18.15)	20.85 (18.15–25.54)	31.36 (29.74–83.72)	83.72	
**MEP**										0.413
All	257	90.3	10.83 (8.61–13.63)	ND	5.54 (4.12–6.32)	11.48 (10.02–13.52)	30.47 (21.78–36.92)	170.77 (120.72–289.87)	4746.88	
A	60	86.7	10.59 (6.24–17.97)	ND	5.76 (2.15–9.80)	13.27 (9.80–19.68)	32.23 (19.68–39.61)	176.10 (61.98–1805.85)	1805.85	
B	40	92.5	12.17 (6.82–21.72)	ND	5.35 (2.51–8.29)	11.52 (6.91–22.49)	31.67 (18.63–84.24)	202.80 (84.24–430.66)	430.66	
C	59	94.9	14.62 (9.52–22.47)	ND	8.15 (3.98–9.73)	12.51 (9.76–21.73)	39.35 (22.90–58.27)	92.14 (62.94–4746.88)	4746.88	
D	38	89.5	9.29 (4.90–17.60)	ND	4.18 (2.07–6.30)	10.52 (5.75–15.82)	26.38 (13.52–39.85)	139.43 (39.85–1673.19)	1673.19	
E	60	88.3	8.41 (5.16–13.72)	ND	4.87 (2.10–6.77)	10.24 (6.77–13.03)	18.58 (13.03–31.99)	160.40 (107.94–876.81)	876.81	
**MBzP**										<0.001 ***
All	257	42.8	0.76 (0.59–0.97)	ND	ND (ND-ND)	ND (ND-ND)	4.39 (3.31–5.78)	16.16 (12.76–32.92)	589.17	
A	60	65.0	2.20 (1.24–3.92)	ND	ND (ND-2.05)	3.79 (2.05–5.58)	9.17 (5.58–11.63)	70.28 (19.08–589.17)	589.17	
B	40	12.5	0.24 (0.16–0.35)	ND	ND (ND-ND)	ND (ND-ND)	ND (ND-ND)	3.52 (ND-26.01)	26.01	
C	59	45.8	0.72 (0.45–1.17)	ND	ND (ND-ND)	ND (ND-2.34)	3.09 (2.43–4.66)	14.29 (8.33–84.25)	84.25	
D	38	15.8	0.26 (0.17–0.40)	ND	ND (ND-ND)	ND (ND-ND)	ND (ND-3.37)	4.41 (3.37–13.12)	13.12	
E	60	55.0	1.17 (0.71–1.95)	ND	ND (ND-ND)	2.17 (ND-4.26)	7.07 (4.26–10.13)	15.15 (11.17–32.92)	32.92	
**MiBP**										0.003 **
All	257	94.6	11.04 (9.19–13.26)	ND	6.00 (5.12–7.12)	9.95 (8.77–11.72)	22.07 (18.11–26.90)	163.88 (98.98–222.38)	528.62	
A	60	85.0	12.21 (6.95–21.45)	ND	7.81 (ND-9.65)	16.33 (9.65–29.07)	36.19 (29.07–61.52)	307.14 (98.98–528.62)	528.62	
B	40	100	12.22 (9.47–15.77)	2.73	7.82 (4.58–8.63)	9.48 (8.52–15.36)	21.32 (11.72–26.80)	40.97 (26.80–117.90)	117.90	
C	59	93.2	7.18 (4.99–10.32)	ND	5.15 (3.64–5.59)	7.82 (5.68–8.65)	12.48 (8.70–14.04)	48.85 (20.87–209.63)	209.63	
D	38	97.4	15.03 (9.25–24.41)	ND	6.09 (3.97–9.84)	12.17 (8.39–19.25)	33.63 (17.93–95.64)	153.16 (95.64–420.27)	420.27	
E	60	100	11.72 (9.08–15.12)	2.29	6.69 (4.67–7.80)	10.10 (7.80–13.72)	17.63 (13.72–21.76)	114.64 (22.35–310.12)	310.12	
**MnBP**										0.003 **
All	257	97.3	17.63 (15.08–20.60)	ND	9.75 (8.38–11.21)	18.12 (15.32–19.73)	29.26 (27.45–34.28)	176.20 (103.51–228.57)	1218.8	
A	60	88.3	15.71 (9.36–26.38)	ND	10.06 (6.09–12.70)	21.15 (12.70–29.04)	35.14 (29.04–86.84)	183.74 (109.11–1218.8)	1218.8	
B	40	100	19.16 (14.53–25.26)	3.68	11.24 (6.91–15.79)	18.88 (14.38–23.01)	28.54 (20.94–37.23)	46.81 (37.23–425.77)	425.77	
C	59	100	12.69 (10.58–15.22)	3.00	8.27 (7.05–9.75)	11.62 (9.98–15.13)	18.36 (15.13–21.89)	30.89 (27.66–186.05)	186.05	
D	38	100	24.42 (16.66–35.81)	3.52	9.01 (5.54–19.72)	25.47 (17.49–31.81)	36.01 (28.12–99.10)	156.33 (99.10–552.53)	552.53	
E	60	100	21.02 (16.39–26.95)	4.80	11.87 (7.14–14.36)	18.17 (14.36–23.11)	29.28 (23.11–37.81)	194.68 (38.46–379.87)	379.87	
**MEHP**										<0.001 ***
All	257	82.1	17.10 (12.33–23.71)	ND	6.87 (2.94–8.95)	18.95 (15.57–28.10)	147.92 (133.73–170.30)	342.11 (266.53–510.44)	37,009	
A	60	46.7	2.03 (0.93–4.40)	ND	ND (ND-ND)	ND (ND-14.33)	29.01 (14.33–89.52)	245.69 (137.50–450.07)	450.07	
B	40	100	18.06 (12.77–25.53)	4.61	9.03 (6.87–11.05)	14.43 (10.12–17.37)	24.40 (15.71–101.09)	135.87 (101.09–395.58)	395.58	
C	59	89.8	18.42 (10.86–31.26)	ND	9.18 (5.65–11.36)	18.11 (11.39–32.42)	128.65 (38.02–143.91)	161.95 (147.92–309.86)	309.86	
D	38	78.9	5.82 (2.85–11.87)	ND	2.56 (ND-8.00)	9.57 (5.46–16.03)	20.06 (13.19–27.29)	129.36 (27.29–147.80)	147.80	
E	60	100	255.7 (193.2–338.6)	39.9	156.7 (139.3–172.0)	208.9 (172.0–223.2)	287.1 (223.2–355.9)	970.2 (384.9–37,009)	37,009	
**MEHHP**										0.552
All	257	98.4	26.24 (22.56–30.52)	ND	14.96 (12.78–16.70)	24.62 (21.90–25.91)	46.67 (35.33–84.23)	186.76 (164.11–215.20)	680.01	
A	60	93.3	23.35 (14.54–37.50)	ND	14.11 (6.73–18.94)	26.79 (18.94–36.19)	79.30 (36.19–138.29)	191.05 (175.39–221.48)	221.48	
B	40	100	26.97 (19.94–36.49)	6.10	14.86 (10.05–20.18)	24.16 (16.74–27.73)	33.37 (26.51–85.24)	128.84 (85.24–680.01)	680.01	
C	59	100	25.17 (19.96–31.72)	2.79	16.08 (13.10–18.58)	24.90 (19.78–26.80)	33.03 (27.95–38.35)	156.49 (75.29–263.28)	263.28	
D	38	100	23.28 (16.29–33.27)	2.05	12.17 (7.12–15.84)	20.64 (14.30–26.24)	35.08 (23.19–96.92)	172.75 (96.92–259.08)	259.08	
E	60	100	32.54 (25.07–42.23)	4.56	16.65 (10.81–18.88)	24.47 (18.88–35.10)	85.97 (35.10–125.73)	180.32 (126.52–201.20)	201.20	
**MEOHP**										<0.001 ***
All	257	92.6	10.56 (8.84–12.62)	ND	7.07 (5.44–8.64)	14.36 (11.94–16.11)	23.92 (20.72–27.11)	62.04 (37.85–108.89)	121.55	
A	60	86.7	12.02 (7.34–19.67)	ND	9.68 (2.04–13.85)	22.30 (13.85–26.70)	32.42 (26.70–48.04)	109.32 (61.81–121.55)	121.55	
B	40	77.5	3.67 (1.87–6.05)	ND	2.58 (ND-4.28)	5.25 (3.52–8.94)	14.12 (6.39–20.46)	26.89 (20.46–38.97)	38.97	
C	59	98.3	14.61 (11.66–18.31)	ND	10.63 (8.29–13.65)	16.79 (14.52–19.24)	21.87 (19.27–27.35)	34.41 (31.93–93.82)	93.82	
D	38	97.4	10.58 (7.46–15.01)	ND	6.72 (4.27–9.08)	11.29 (8.78–15.26)	17.79 (13.16–25.11)	40.85 (25.11–109.30)	109.30	
E	60	100	14.44 (11.59–17.99)	2.68	8.25 (5.57–9.86)	14.18 (9.86–19.18)	24.64 (19.18–32.35)	85.14 (34.31–118.38)	118.38	
**MECPP**										<0.001 ***
All	257	97.3	33.29 (27.90–39.72)	ND	16.83 (14.75–19.56)	29.91 (25.98–31.77)	88.37 (62.08–117.64)	296.82 (243.38–380.21)	621.22	
A	60	88.3	30.20 (16.70–54.61)	ND	15.72 (5.36–26.95)	39.08 (26.95–77.19)	185.27 (77.19–245.94)	336.72 (277.63–523.22)	523.22	
B	40	100	23.84 (18.38–30.91)	6.63	14.91 (9.90–19.63)	21.74 (17.28–26.41)	31.60 (24.98–35.77)	109.45 (35.77–621.22)	621.22	
C	59	100	27.48 (21.72–34.77)	2.68	15.93 (11.35–20.82)	25.98 (20.84–30.58)	34.63 (30.63–64.08)	210.12 (82.48–256.64)	256.64	
D	38	100	29.16 (20.47–41.53)	3.93	15.00 (10.15–19.66)	24.14 (18.82–30.28)	32.83 (28.22–101.68)	279.61 (101.68–339.74)	339.74	
E	60	100	60.19 (45.05–80.42)	8.21	23.67 (16.95–30.62)	80.77 (30.62–98.97)	153.63 (98.97–210.92)	339.18 (234.05–504.30)	504.30	
**MCMHP**										<0.001 ***
All	257	94.6	10.64 (8.90–12.73)	ND	5.59 (4.45–6.34)	11.67 (9.09–14.37)	24.25 (21.25–29.44)	98.62 (60.50–169.48)	393.04	
A	60	90.0	13.12 (8.47–20.31)	ND	10.41 (7.63–14.37)	19.67 (14.37–24.97)	33.85 (24.97–36.38)	62.85 (38.18–364.15)	364.15	
B	40	95.0	4.80 (3.41–6.74)	ND	3.44 (2.53–3.69)	5.38 (3.65–6.35)	7.44 (6.07–10.26)	13.16 (10.26–109.03)	109.03	
C	59	96.6	8.55 (6.20–11.79)	ND	4.72 (3.71–5.21)	7.46 (5.24–9.50)	16.37 (10.59–28.35)	61.50 (29.44–240.95)	240.95	
D	38	94.7	20.91 (11.80–37.05)	ND	10.29 (4.04–16.77)	22.89 (14.56–31.99)	37.14 (28.77–114.52)	322.69 (114.52–393.04)	393.04	
E	60	96.7	11.88 (8.79–16.06)	ND	7.37 (4.79–9.01)	13.84 (9.01–17.54)	21.31 (17.54–28.27)	81.77 (34.39–101.69)	101.69	
**MiNP**										<0.001 ***
All	257	36.6	0.79 (0.59–1.04)	ND	ND (ND-ND)	ND (ND-ND)	8.37 (4.29–11.13)	37.46 (28.67–134.00)	462.14	
A	60	70.0	3.63 (2.01–6.53)	ND	ND (ND-2.73)	8.67 (2.73–14.44)	18.83 (14.44–24.87)	42.47 (28.82–230.50)	230.50	
B	40	7.5	0.19 (0.14–0.26)	ND	ND (ND-ND)	ND (ND-ND)	ND (ND-ND)	2.36 (ND-7.57)	7.57	
C	59	11.9	0.26 (0.17–0.41)	ND	ND (ND-ND)	ND (ND-ND)	ND (ND-ND)	10.68 (ND-346.37)	346.37	
D	38	18.4	0.30 (0.18–0.50)	ND	ND (ND-ND)	ND (ND-ND)	ND (ND-5.59)	8.56 (5.59–14.67)	14.67	
E	60	58.3	2.33 (1.22–4.48)	ND	ND (ND-ND)	4.24 (ND-11.13)	16.99 (11.13–30.64)	118.47 (37.67–462.14)	462.14	
**ΣDEHPm (nmole/mL) ^c^**										<0.001 ***
All	257		0.54 (0.46–0.63)	<0.01	0.25 (0.22–0.29)	0.56 (0.44–0.67)	1.17 (1.00–1.42)	2.96 (2.57–4.26)	133.14	
A	60		0.43 (0.29–0.64)	<0.01	0.22 (0.16–0.27)	0.43 (0.27–0.71)	1.52 (0.71–1.99)	2.67 (2.16–4.74)	4.74	
B	40		0.31 (0.23–0.41)	0.09	0.17 (0.12–0.22)	0.27 (0.20–0.37)	0.51 (0.32–0.69)	1.33 (0.69–5.24)	5.24	
C	59		0.46 (0.37–0.57)	0.03	0.29 (0.23–0.33)	0.52 (0.33–0.69)	0.74 (0.70–0.93)	1.72 (1.07–2.00)	2.00	
D	38		0.36 (0.25–0.52)	0.03	0.20 (0.13–0.25)	0.30 (0.23–0.38)	0.56 (0.37–1.17)	2.83 (1.17–4.07)	4.07	
E	60		1.52 (1.16–2.00)	0.20	0.87 (0.67–1.04)	1.22 (1.04–1.43)	2.05 (1.43–2.67)	4.96 (2.98–133.14)	133.14	
**ΣDBPm (nmole/mL) ^d^**										<0.001 ***
All	257		0.14 (0.12–0.17)	<0.01	0.08 (0.07–0.09)	0.14 (0.12–0.15)	0.23 (0.22–0.27)	1.50 (0.90–2.42)	5.93	
A	60		0.16 (0.09–0.26)	<0.01	0.09 (0.05–0.13)	0.21 (0.13–0.25)	0.41 (0.25–0.70)	2.78 (0.90–5.93)	5.93	
B	40		0.15 (0.12–0.19)	0.04	0.10 (0.06–0.12)	0.14 (0.11–0.17)	0.22 (0.16–0.28)	0.72 (0.28–2.28)	2.28	
C	59		0.10 (0.08–0.12)	0.01	0.06 (0.05–0.07)	0.09 (0.07–0.12)	0.15 (0.12–0.19)	0.52 (0.20–1.75)	1.75	
D	38		0.20 (0.14–0.30)	0.02	0.09 (0.04–0.14)	0.20 (0.11–0.27)	0.44 (0.22–0.79)	1.61 (0.79–3.34)	3.34	
E	60		0.15 (0.12–0.20)	0.03	0.09 (0.05–0.11)	0.14 (0.11–0.16)	0.22 (0.16–0.25)	1.43 (0.57–2.42)	2.42	

^a^ Limit of detection (LOD), ND was calculated as half LOD. The LODs for MMP, MEP, MiBP, MnBP, MBzP, MEHP, MEHHP, MEOHP, MECPP, MCMHP, and MiNP were 0.3, 0.3, 1.0, 1.0, 0.3, 0.7, 0.3, 0.3, 0.3, 0.1, and 0.1 ng/mL, respectively. ^b^ Comparison of school groups using the Kruskal–Wallis test. * *p* < 0.05, ** *p* < 0.01, *** *p* < 0.001. ^c^ ΣDEHPm = sum of molar concentrations of MEHP + MEHHP + MEOHP + MECPP + MCMHP. ^d^ ΣDBPm = sum of molar concentrations of MiBP + MnBP.

**Table 3:** Hazard quotients and the hazard index for participants by elementary school group (*n* = 257).

Index	*n*	>1 (%)	GM (95%CI)	Min	Selected Percentiles				Max	*p*-Value ^a^
					25th (95% CI)	50th (95% CI)	75th (95% CI)	95th (95% CI)		
**HQ_DEHP_**										<0.001 ***
All	257	1.6	0.11 (0.10–0.13)	<0.01	0.05 (0.05–0.06)	0.11 (0.09–0.13)	0.24 (0.20–0.27)	0.55 (0.47–0.70)	53.51	
A	60	0	0.08 (0.06–0.12)	<0.01	0.05 (0.03–0.07)	0.11 (0.07–0.15)	0.21 (0.15–0.27)	0.43 (0.30–0.68)	0.68	
B	40	0	0.07 (0.06–0.08)	0.01	0.05 (0.03–0.05)	0.06 (0.05–0.09)	0.09 (0.08–0.13)	0.17 (0.13–0.51)	0.51	
C	59	0	0.09 (0.07–0.11)	0.01	0.05 (0.04–0.05)	0.08 (0.06–0.11)	0.15 (0.11–0.22)	0.30 (0.24–0.55)	0.55	
D	38	0	0.08 (0.06–0.11)	0.03	0.05 (0.03–0.05)	0.06 (0.05–0.09)	0.13 (0.08–0.27)	0.38 (0.27–0.93)	0.93	
E	60	6.7	0.33 (0.25–0.43)	0.05	0.18 (0.14–0.23)	0.27 (0.23–0.36)	0.46 (0.36–0.56)	1.41 (0.65–53.51)	53.51	
**HQ_DnBP_**										<0.001 ***
All	257	1.2	0.05 (0.04–0.05)	<0.01	0.03 (0.03–0.03)	0.04 (0.04–0.05)	0.07 (0.06–0.08)	0.32 (0.20–0.51)	1.42	
A	60	1.7	0.04 (0.02–0.06)	<0.01	0.03 (0.02–0.04)	0.06 (0.04–0.06)	0.13 (0.06–0.16)	0.32 (0.17–1.04)	1.04	
B	40	2.5	0.05 (0.04–0.07)	0.01	0.03 (0.03–0.04)	0.05 (0.04–0.06)	0.07 (0.05–0.10)	0.14 (0.10–1.16)	1.16	
C	59	0	0.03 (0.03–0.04)	0.01	0.02 (0.02–0.03)	0.03 (0.03–0.03)	0.04 (0.03–0.05)	0.07 (0.05–0.34)	0.34	
D	38	2.6	0.07 (0.05–0.10)	0.02	0.03 (0.03–0.04)	0.06 (0.04–0.08)	0.10 (0.08–0.17)	0.50 (0.17–1.42)	1.42	
E	60	0	0.06 (0.05–0.07)	0.02	0.04 (0.03–0.04)	0.05 (0.04–0.06)	0.07 (0.06–0.13)	0.25 (0.17–0.96)	0.96	
**HQ_DiBP_**										<0.001 ***
All	257	0.8	0.04 (0.03–0.04)	<0.01	0.02 (0.02–0.02)	0.03 (0.03–0.04)	0.07 (0.06–0.09)	0.42 (0.22–0.59)	2.78	
A	60	1.7	0.04 (0.02–0.06)	<0.01	0.02 (<0.01–0.04)	0.06 (0.04–0.08)	0.12 (0.08–0.15)	0.59 (0.28–1.30)	1.30	
B	40	0	0.04 (0.03–0.05)	0.01	0.02 (0.02–0.04)	0.04 (0.04–0.05)	0.06 (0.04–0.09)	0.19 (0.09–0.27)	0.27	
C	59	0	0.02 (0.02–0.03)	<0.01	0.01 (0.01–0.02)	0.02 (0.02–0.03)	0.03 (0.03–0.04)	0.13 (0.06–0.45)	0.45	
D	38	2.6	0.05 (0.03–0.08)	<0.01	0.02 (0.01–0.03)	0.04 (0.03–0.06)	0.09 (0.05–0.27)	0.60 (0.27–2.78)	2.78	
E	60	0	0.04 (0.03–0.05)	0.01	0.02 (0.02–0.03)	0.03 (0.03–0.04)	0.04 (0.04–0.09)	0.20 (0.13–0.84)	0.84	
**HQ_DiNP_**										<0.001 ***
All	257	5.4	0.02 (0.01–0.03)	<0.01	<0.01 (<0.01–<0.01)	0.01 (0.01–0.01)	0.16 (0.09–0.27)	1.12 (0.61–2.84)	7.44	
A	60	6.7	0.08 (0.05–0.15)	<0.01	0.01 (<0.01–0.06)	0.18 (0.06–0.36)	0.47 (0.36–0.55)	1.09 (0.74–6.15)	6.15	
B	40	0	<0.01 (<0.01–0.01)	<0.01	<0.01 (<0.01–<0.01)	<0.01 (<0.01–0.01)	0.01 (0.01–0.01)	0.06 (0.01–0.09)	0.09	
C	59	3.4	0.01 (<0.01–0.01)	<0.01	<0.01 (<0.01–<0.01)	<0.01 (<0.01–<0.01)	0.01 (0.01–0.01)	0.21 (0.01–7.02)	7.02	
D	38	0	0.01 (<0.01–0.01)	<0.01	<0.01 (<0.01–<0.01)	<0.01 (<0.01–0.01)	0.01 (0.01–0.09)	0.22 (0.09–0.49)	0.49	
E	60	13.3	0.06 (0.03–0.11)	<0.01	0.01 (<0.01–0.01)	0.09 (0.01–0.23)	0.42 (0.23–0.64)	2.88 (1.41–7.44)	7.44	
**HQ_BBzP_**										<0.001 ***
All	257	0	4.4 × 10^−4^ (3.6 × 10^−4^–5.7 × 10^−4^)	3.1 × 10^−5^	9.4 × 10^−5^ (7.4 × 10^−5^–1.1 × 10^−4^)	1.9 × 10^−4^ (1.5 × 10^−4^–3.2 × 10^−4^)	2.5 × 10^−3^ (1.7 × 10^−3^–3.1 × 10^−3^)	9.2 × 10^−3^ (6.7 × 10^−3^–1.5 × 10^−2^)	2.2 × 10^−1^	
A	60	0	1.2 × 10^−3^ (7.3 × 10^−4^–2.1 × 10^−3^)	4.8 × 10^−5^	1.5 × 10^−4^ (9.6 × 10^−5^–6.5 × 10^−4^)	2.1 × 10^−3^ (6.5 × 10^−4^–3.1 × 10^−3^)	5.8 × 10^−3^ (3.1 × 10^−3^–7.2 × 10^−3^)	3.2 × 10^−2^ (9.2 × 10^−3^–2.2 × 10^−1^)	2.2 × 10^−1^	
B	40	0	1.5 × 10^−4^ (1.0 × 10^−4^–2.2 × 10^−4^)	3.2 × 10^−5^	7.3 × 10^−5^ (6.2 × 10^−5^–9.2 × 10^−5^)	1.1 × 10^−4^ (8.7 × 10^−5^–1.3 × 10^−4^)	1.7 × 10^−4^ (1.3 × 10^−4^–3.0 × 10^−4^)	2.6 × 10^−3^ (3.0 × 10^−4^–6.1 × 10^−3^)	6.1 × 10^−3^	
C	59	0	3.9 × 10^−4^ (2.4 × 10^−4^–6.3 × 10^−4^)	4.0 × 10^−5^	7.1 × 10^−5^ (5.9 × 10^−5^–1.1 × 10^−4^)	1.7 × 10^−4^ (1.1 × 10^−4^–9.0 × 10^−4^)	1.7 × 10^−3^ (1.2 × 10^−3^–2.7 × 10^−3^)	6.6 × 10^−3^ (3.6 × 10^−3^–2.8 × 10^−2^)	2.8 × 10^−2^	
D	38	0	1.7 × 10^−4^ (1.1 × 10^−4^–2.6 × 10^−4^)	3.3 × 10^−5^	7.7 × 10^−5^ (5.3 × 10^−5^–9.4 × 10^−5^)	1.1 × 10^−4^ (9.0 × 10^−5^–1.6 × 10^−4^)	2.1 × 10^−4^ (1.4 × 10^−4^–9.3 × 10^−4^)	3.0 × 10^−3^ (9.3 × 10^−4^–5.3 × 10^−3^)	5.3 × 10^−3^	
E	60	0	7.2 × 10^−4^ (4.5 × 10^−4^–1.2 × 10^−3^)	3.1 × 10^−5^	1.3 × 10^−4^ (9.2 × 10^−5^–1.9 × 10^−4^)	9.7 × 10^−4^ (1.9 × 10^−4^–1.8 × 10^−3^)	4.2 × 10^−3^(1.8 × 10^−3^–5.6 × 10^−3^)	9.4 × 10^−3^ (6.4 × 10^−3^–2.7 × 10^−2^)	2.7 × 10^−2^	
**HQ_DEP_**										0.493
All	257	0	6.4 × 10^−4^ (5.1 × 10^−4^–7.9 × 10^−4^)	5.1 × 10^−6^	3.2 × 10^−4^ (2.6 × 10^−4^–3.7 × 10^−4^)	6.5 × 10^−4^ (5.6 × 10^−4^–7.9 × 10^−4^)	1.6 × 10^−3^ (1.3 × 10^−3^–2.3 × 10^−3^)	7.7 × 10^−3^ (5.7 × 10^−3^–1.4 × 10^−2^)	4.3 × 10^−1^	
A	60	0	5.9 × 10^−4^ (3.5 × 10^−4^–1.0 × 10^−3^)	5.1 × 10^−6^	2.8 × 10^−4^ (1.2 × 10^−4^–5.5 × 10^−4^)	8.3 × 10^−4^ (5.5 × 10^−4^–1.3 × 10^−3^)	1.6 × 10^−3^ (1.3 × 10^−3^–2.8 × 10^−3^)	6.8 × 10^−3^ (3.5 × 10^−3^–5.1 × 10^−2^)	5.1 × 10^−2^	
B	40	0	7.7 × 10^−4^ (4.4 × 10^−4^–1.3 × 10^−3^)	6.2 × 10^−6^	3.6 × 10^−4^ (2.2 × 10^−4^–5.9 × 10^−4^)	7.8 × 10^−4^ (4.6 × 10^−4^–1.2 × 10^−3^)	2.1 × 10^−3^ (1.1 × 10^−3^–4.1 × 10^−3^)	8.0 × 10^−3^ (4.1 × 10^−3^–3.3 × 10^−2^)	3.3 × 10^−2^	
C	59	0	7.9 × 10^−4^ (5.3 × 10^−4^–1.2 × 10^−3^)	2.5 × 10^−5^	3.7 × 10^−4^ (3.1 × 10^−4^–4.4 × 10^−4^)	6.7 × 10^−4^ (4.7 × 10^−4^–9.7 × 10^−4^)	2.2 × 10^−3^ (1.1 × 10^−3^–2.9 × 10^−3^)	5.1 × 10^−3^ (3.1 × 10^−3^–4.3 × 10^−1^)	4.3 × 10^−1^	
D	38	0	6.0 × 10^−4^ (3.2 × 10^−4^–1.1 × 10^−3^)	5.7 × 10^−6^	2.6 × 10^−4^ (1.6 × 10^−4^–4.4 × 10^−4^)	5.2 × 10^−4^ (3.4 × 10^−4^–9.8 × 10^−4^)	1.2 × 10^−3^ (8.2 × 10^−4^–4.7 × 10^−3^)	7.9 × 10^−3^ (4.7 × 10^−3^–7.8 × 10^−2^)	7.8 × 10^−2^	
E	60	0	5.1 × 10^−4^ (3.3 × 10^−4^–8.0 × 10^−4^)	1.1 × 10^−5^	3.1 × 10^−4^ (1.3 × 10^−4^–3.6 × 10^−4^)	5.2 × 10^−4^ (3.6 × 10^−4^–6.8 × 10^−4^)	9.6 × 10^−4^ (6.8 × 10^−4^–2.0 × 10^−3^)	7.8 × 10^−3^ (4.2 × 10^−3^–3.6 × 10^−2^)	3.6 × 10^−2^	
**HI_hep_^b^**										<0.001 ***
All	257	13.2	0.33 (0.28–0.38)	<0.01	0.15 (0.13–0.17)	0.32 (0.26–0.41)	0.66 (0.58–0.75)	1.79 (1.37–2.67)	133.78	
A	60	8.3	0.30 (0.21–0.43)	<0.01	0.19 (0.10–0.26)	0.43 (0.26–0.49)	0.61 (0.49–0.84)	1.78 (0.94–2.67)	2.67	
B	40	2.5	0.17 (0.14–0.21)	0.03	0.12 (0.09–0.14)	0.15 (0.13–0.22)	0.23 (0.20–0.32)	0.44 (0.32–1.29)	1.29	
C	59	3.4	0.24 (0.19–0.30)	0.02	0.13 (0.10–0.17)	0.25 (0.18–0.28)	0.42 (0.29–0.60)	0.79 (0.69–2.47)	2.47	
D	38	5.3	0.22 (0.17–0.29)	0.07	0.13 (0.08–0.16)	0.17 (0.14–0.23)	0.33 (0.19–0.68)	0.96 (0.68–2.33)	2.33	
E	60	40.0	0.96 (0.73–1.28)	0.13	0.52 (0.46–0.61)	0.72 (0.61–1.09)	1.37 (1.09–1.69)	3.85 (1.85–133.78)	133.78	
**HI_rep_**										<0.001 ***
All	257	7.8	0.24 (0.21–0.28)	<0.01	0.14 (0.12–0.15)	0.24 (0.21–0.27)	0.41 (0.37–0.48)	1.19 (0.94–1.97)	53.81	
A	60	11.7	0.22 (0.15–0.32)	<0.01	0.15 (0.10–0.23)	0.32 (0.23–0.39)	0.48 (0.39–0.54)	1.19 (0.70–1.97)	1.97	
B	40	2.5	0.18 (0.15–0.23)	0.04	0.12 (0.10–0.15)	0.17 (0.13–0.21)	0.25 (0.20–0.29)	0.60 (0.29–1.53)	1.53	
C	59	0	0.16 (0.13–0.19)	0.05	0.09 (0.07–0.11)	0.15 (0.11–0.20)	0.27 (0.20–0.30)	0.41 (0.35–0.84)	0.84	
D	38	7.9	0.26 (0.19–0.35)	0.06	0.14 (0.11–0.17)	0.18 (0.15–0.31)	0.36 (0.29–0.62)	2.11 (0.62–3.16)	3.16	
E	60	15.0	0.47 (0.36–0.61)	0.12	0.25 (0.21–0.29)	0.41 (0.29–0.49)	0.62 (0.49–0.79)	1.97 (1.03–53.81)	53.81	

Abbreviations: confidence interval (CI), hazard quotient (HQ), hazard index (HI). ^a^ Comparison of different school groups using the Kruskal–Wallis test. * *p* < 0.05, ** *p* < 0.01, *** *p* < 0.001. ^b^ RfDs proposed by the U.S. EPA and EFSA for DEHP, BBzP, and DiNP are 20, 200, and 150 μg/kg/day, respectively.

**Table 4:** Comparison of estimated daily phthalate intake (μg/kg/day), hazard quotients, and hazard indices for participants (*n* = 257) from different elementary schools.

Daily Intakes/Index	A (*n* = 60)	B (*n* = 40)	C (*n* = 59)	D (*n* = 38)	E (*n* = 60)	*p* Value ^a^
Mean ^b^						
DEHP	7.48	4.30	5.87	6.45	80.44	0.011 *
DnBP	0.99	0.94	0.39	1.41	0.91	0.924
DiBP	1.37	0.58	0.43	1.76	0.68	0.358
DEP	1.24	1.25	4.28	1.79	1.04	0.864
DMP	0.39	0.57	0.67	1.28	0.50	0.013 *
HQ_DEHP_	0.15	0.09	0.12	0.13	1.61	0.011 *
HQ_DnBP_	0.10	0.09	0.04	0.14	0.09	0.924
HQ_DiBP_	0.14	0.06	0.04	0.18	0.07	0.358
HQ_DEP_	0.002	0.003	0.009	0.004	0.002	0.864
HI_hep_	0.53	0.22	0.35	0.34	4.22	0.010 *
HI_rep_	0.39	0.24	0.20	0.45	1.77	0.014 *

^a^ ANCOVA for mean. * *p* < 0.05, ** *p* < 0.01, *** *p* < 0.001. ^b^ ANCOVA adjusted for age, sex, passive smoking exposure, BMI, father ever employed at petrochemical complex, and home location close to a main road.

## Data Availability

Data are contained within the article.

## Supplementary Materials

The following supporting information can be downloaded at: https://www.mdpi.com/article/10.3390/toxics11010057/s1, Table S1: Estimated daily intake (μg/kg/day) for seven PAEs in participants by school group (*n* = 257); Table S2: Comparison of urinary phthalate levels for participants (*n* = 257) from different schools by mean after adjusting for covariance; Table S3: Comparison of urinary phthalate levels for participants (*n* = 257) from different schools by median; Table S4: Comparison of urinary phthalate levels for participants (*n* = 257) from different schools by mean after adjusting for covariance; Table S5: Comparison of estimated daily phthalate intake (μg/kg/day) for participants (*n* = 257) from different schools; Table S6: Comparison of hazard quotients and hazard index by tolerable daily intake for participants (*n* = 257) from different schools; Table S7: Annual levels of air vinyl chloride monomers (VCM) and 1,1-dichloroethane at petrochemical complex and surrounding region (schools and community) from May 2012 to June 2014 per local EPA of Yun-Lin County, Taiwan; Figure S1: Locations of air VCM and 1,1-dichloroethane monitoring sites inside (1–3) and outside (4–7) petrochemical complex (No. 6 Naphtha Cracking Complex) in central Taiwan.
