# Supplementary material for: Exposure Characteristics and Cumulative Risk Assessment for Phthalates in Children Living near a Petrochemical Complex
Source: Toxics. 2023 Jan 6;11(1):57. doi: 10.3390/toxics11010057 (PMC9865072; doi:10.3390/toxics11010057)
Supplement: Supplementary file 1 [file toxics-11-00057-s001.zip › toxics-2034684-supplementary.pdf]

Supporting Information

For

Exposure characteristics and cumulative risk assessment of phthalates in children living near a petrochemical complex

Chih-Wen Wang [1,2,3,†], Po-Keng Cheng [4,†], Vinoth Kumar Ponnusamy [3,5], Hung-Che Chiang [6], Wan-Ting Chang [7] and Po-Chin Huang [3,6,7,*]

1 Division of Hepatobiliary, Department of Internal Medicine, Kaohsiung Medical University Hospital, Kaohsiung Medical University, Kaohsiung 701, Taiwan
2 Department of Internal Medicine, Kaohsiung Municipal Siaogang Hospital, Kaohsiung Medical University, Kaohsiung 701, Taiwan
3 Research Center for Precision Environmental Medicine, Kaohsiung Medical University, Kaohsiung 701, Taiwan
4 Department of Finance and Cooperative Management, National Taipei University, New Taipei City 237, Taiwan
5 Department of Medicinal and Applied Chemistry, Kaohsiung Medical University, Kaohsiung 807, Taiwan
6 Department of Medical Research, China Medical University Hospital, China Medical University, Taichung 404, Taiwan
7 National Institute of Environmental Health Sciences, National Health Research Institutes, Miaoli 350, Taiwan
* Correspondence: pchuang@nhri.edu.tw; Tel.: +886-37-206166 (ext. 38507)
† These authors contributed equally to this work.

No. of figures: 1

No. of Tables: 7

No. of pages: 14

Supplementary Table S1. Estimated daily intake (μg/kg/day) for seven phthalates in participants by school group (N = 257).

	N	> TDI (%) [a]	GM (95%CI)	Min	Selected percentiles				Max	*p*-value [b]
					**25th (95%CI)**	**50th (95%CI)**	**75th (95%CI)**	**95th (95%CI)**		
**DEHP**										**<0.001*****
All	257	1.6	5.60 (4.84-6.48)	0.03	2.64 (2.34-2.91)	5.54 (4.57-6.37)	11.84 (10.21-13.52)	27.41 (23.42-34.83)	2675.62	
School A	60	0	4.24 (2.95-6.09)	0.03	2.32 (1.39-3.67)	5.62 (3.67-7.65)	10.58 (7.65-13.40)	21.74 (14.76-34.07)	34.07	
School B	40	0	3.42 (2.80-4.19)	0.67	2.40 (1.73-2.74)	2.87 (2.57-4.39)	4.64 (3.96-6.31)	8.73 (6.31-25.29)	25.29	
School C	59	0	4.30 (3.48-5.31)	0.41	2.35 (1.79-2.67)	4.18 (2.96-5.47)	7.49 (5.53-11.07)	15.04 (11.84-27.40)	27.40	
School D	38	0	4.08 (3.06-5.44)	1.27	2.34 (1.68-2.72)	3.22 (2.50-4.35)	6.51 (3.76-13.45)	19.24 (13.45-46.49)	46.49	
School E	60	6.7	16.30 (12.25-21.67)	2.49	8.89 (6.89-11.43)	13.75 (11.43-18.09)	23.09 (18.09-28.14)	70.48 (32.28-2675.62)	2675.62	
**DnBP**										**<0.001*****
All	257	1.2	0.47 (0.40-0.54)	<0.01	0.30 (0.27-0.31)	0.43 (0.39-0.47)	0.72 (0.62-0.84)	3.21 (1.98-5.08)	14.16	
School A	60	1.7	0.40 (0.25-0.63)	<0.01	0.28 (0.19-0.40)	0.55 (0.40-0.62)	1.34 (0.62-1.57)	3.22 (1.67-10.44)	10.44	
School B	40	2.5	0.54 (0.42-0.71)	0.12	0.35 (0.25-0.42)	0.48 (0.40-0.62)	0.74 (0.53-1.00)	1.43 (1.00-11.61)	11.61	
School C	59	0	0.31 (0.26-0.36)	0.07	0.24 (0.16-0.28)	0.31 (0.28-0.33)	0.39 (0.34-0.46)	0.67 (0.49-3.41)	3.41	
School D	38	2.6	0.70 (0.51-0.98)	0.18	0.34 (0.30-0.39)	0.56 (0.37-0.80)	1.04 (0.78-1.66)	4.97 (1.66-14.16)	14.16	
School E	60	0	0.58 (0.47-0.71)	0.15	0.38 (0.29-0.42)	0.48 (0.42-0.59)	0.66 (0.59-1.31)	2.52 (1.66-9.63)	9.63	
**DiBP**										**<0.001*****
All	257	0.8	0.36 (0.30-0.42)	<0.01	0.20 (0.17-0.23)	0.35 (0.30-0.37)	0.69 (0.56-0.88)	4.25 (2.17-5.91)	27.83	
School A	60	1.7	0.37 (0.22-0.64)	<0.01	0.24 (0.01-0.39)	0.57 (0.39-0.79)	1.22 (0.79-1.49)	5.92 (2.77-13.02)	13.02	
School B	40	0	0.42 (0.33-0.54)	0.10	0.25 (0.17-0.37)	0.41 (0.36-0.49)	0.62 (0.44-0.88)	1.86 (0.88-2.69)	2.69	
School C	59	0	0.21 (0.16-0.29)	0.01	0.13 (0.10-0.17)	0.23 (0.18-0.26)	0.34 (0.28-0.40)	1.26 (0.59-4.51)	4.51	
School D	38	2.6	0.53 (0.33-0.83)	0.02	0.21 (0.15-0.29)	0.42 (0.25-0.56)	0.95 (0.53-2.67)	5.98 (2.67-27.83)	27.83	
School E	60	0	0.39 (0.31-0.49)	0.11	0.23 (0.20-0.26)	0.33 (0.26-0.35)	0.45 (0.35-0.90)	2.00 (1.28-8.38)	8.38	

Supplementary Table S1. (continued)

	N	> TDI (%) [a]	GM (95%CI)	Min	Selected percentiles				Max	*p*-value [b]
					25th (95%CI)	50th (95%CI)	75th (95%CI)	95th (95%CI)		
**DiNP**										**<0.001*****
All	257	5.4	0.95 (0.72-1.26)	0.06	0.15 (0.13-0.19)	0.30 (0.26-0.36)	7.89 (4.28-13.74)	56.15 (30.72-141.85)	371.79	
School A	60	6.7	4.18 (2.30-7.61)	0.06	0.35 (0.16-3.21)	8.94 (3.21-17.82)	23.56 (17.82-27.32)	54.40 (37.01-307.40)	307.40	
School B	40	0	0.25 (0.19-0.33)	0.07	0.14 (0.11-0.19)	0.23 (0.17-0.27)	0.32 (0.25-0.41)	2.99 (0.41-4.28)	4.28	
School C	59	3.4	0.29 (0.19-0.45)	0.08	0.11 (0.10-0.13)	0.17 (0.13-0.23)	0.30 (0.25-0.51)	10.69 (0.62-351.01)	351.01	
School D	38	0	0.40 (0.24-0.66)	0.07	0.15 (0.10-0.19)	0.24 (0.19-0.32)	0.46 (0.30-4.71)	11.02 (4.71-24.49)	24.49	
School E	60	13.3	2.93 (1.53-5.58)	0.06	0.26 (0.16-0.38)	4.35 (0.38-11.61)	21.08 (11.61-32.05)	143.87 (70.26-371.79)	371.79	
**BBzP**										**<0.001*****
All	257	0	0.02 (0.02-0.03)	<0.01	<0.01 (<0.01-0.01)	0.01 (0.01-0.02)	0.12 (0.08-0.16)	0.46 (0.34-0.75)	10.86	
School A	60	0	0.06 (0.04-0.11)	<0.01	0.01 (<0.01-0.03)	0.10 (0.03-0.15)	0.29 (0.15-0.36)	1.58 (0.46-10.86)	10.86	
School B	40	0	0.01 (0.01-0.01)	<0.01	<0.01 (<0.01-<0.01)	0.01 (<0.01-0.01)	0.01 (0.01-0.02)	0.13 (0.02-0.31)	0.31	
School C	59	0	0.02 (0.01-0.03)	<0.01	<0.01 (<0.01-0.01)	0.01 (0.01-0.05)	0.08 (0.06-0.13)	0.33 (0.18-1.42)	1.42	
School D	38	0	0.01 (0.01-0.01)	<0.01	<0.01 (<0.01-<0.01)	0.01 (<0.01-0.01)	0.01 (0.01-0.05)	0.15 (0.05-0.26)	0.26	
School E	60	0	0.04 (0.02-0.06)	<0.01	0.01 (<0.01-0.01)	0.05 (0.01-0.09)	0.21 (0.09-0.28)	0.47 (0.32-1.33)	1.33	
**DEP**										0.493
All	257	0	0.32 (0.26-0.40)	<0.01	0.16 (0.13-0.18)	0.33 (0.28-0.40)	0.81 (0.65-1.17)	3.87 (2.86-7.08)	212.69	
School A	60	0	0.30 (0.18-0.50)	<0.01	0.14 (0.06-0.28)	0.41 (0.28-0.67)	0.81 (0.67-1.40)	3.41 (1.77-25.52)	25.52	
School B	40	0	0.38 (0.22-0.66)	<0.01	0.18 (0.11-0.30)	0.39 (0.23-0.58)	1.04 (0.54-2.04)	4.00 (2.04-16.55)	16.55	
School C	59	0	0.39 (0.26-0.59)	0.01	0.19 (0.16-0.22)	0.34 (0.24-0.48)	1.11 (0.57-1.45)	2.53 (1.56-212.69)	212.69	
School D	38	0	0.30 (0.16-0.55)	<0.01	0.13 (0.08-0.22)	0.26 (0.17-0.49)	0.62 (0.41-2.33)	3.97 (2.33-39.06)	39.06	
School E	60	0	0.26 (0.16-0.40)	0.01	0.15 (0.06-0.18)	0.26 (0.18-0.34)	0.48 (0.34-1.01)	3.90 (2.09-17.95)	17.95	

Supplementary Table S1. (continued)

	N	> TDI (%) [a]	GM (95%CI)	Min	Selected percentiles				Max	*p*-value [b]
					25th (95%CI)	50th (95%CI)	75th (95%CI)	95th (95%CI)		
**DMP**										**<0.001*****
All	257		0.40 (0.34-0.46)	<0.01	0.28 (0.23-0.33)	0.46 (0.41-0.51)	0.71 (0.65-0.82)	1.81 (1.25-3.19)	6.39	
School A	60		0.21 (0.14-0.31)	<0.01	0.16 (0.04-0.24)	0.37 (0.24-0.43)	0.54 (0.43-0.61)	0.84 (0.65-1.83)	1.83	
School B	40		0.46 (0.35-0.60)	0.01	0.33 (0.23-0.38)	0.55 (0.37-0.68)	0.74 (0.60-0.93)	1.08 (0.93-1.38)	1.38	
School C	59		0.45 (0.34-0.60)	<0.01	0.33 (0.21-0.39)	0.57 (0.43-0.68)	0.74 (0.68-0.94)	1.86 (1.03-3.34)	3.34	
School D	38		0.78 (0.53-1.13)	0.01	0.43 (0.33-0.64)	0.86 (0.53-1.00)	1.09 (0.98-3.19)	4.10 (3.19-6.39)	6.39	
School E	60		0.40 (0.34-0.47)	0.10	0.27 (0.21-0.34)	0.41 (0.34-0.45)	0.51 (0.45-0.66)	1.40 (0.77-2.27)	2.27	

Abbreviations: confidence interval (CI), benzyl butyl phthalate (BBzP), di-iso-butyl phthalate (DiBP), di-n-butyl phthalate (DnBP), di-ethyl phthalate (DEP), di-2-ethylhexyl phthalate (DEHP), dimethyl phthalate (DMP), di-iso-nonyl phthalate (DiNP), tolerable daily intake (TDI).

[a] TDIs proposed by the European Food Safety Authority for DEHP, DnBP, DiBP, BBzP, DEP, and DiNP are equal to 50, 10, 10, 50, 500, and 50 µg/kg/day, respectively.

[b] Comparison of school groups using the Kruskal–Wallis test. *$p < 0.05$, **$p < 0.01$, ***$p < 0.001$.

Supplementary Table S2. Comparison of urinary phthalate levels for participants (N = 257) from different schools by mean after adjusting for covariance[a].

	**A (N=60)**	**B (N=40)**	**C (N=59)**	**D (N=38)**	**E (N=60)**	***P* value[b]**
**MMP (ng/mL)**	17.09	20.20	31.47	53.89	17.92	0.088
**MEP (ng/mL)**	62.05	44.01	108.72	67.03	39.28	0.708
**MiBP (ng/mL)**	57.63	17.70	18.41	41.87	24.46	0.122
**MnBP (ng/mL)**	61.59	33.73	17.56	52.34	39.02	0.580
**MEHP (ng/mL)**	44.96	39.71	58.35	24.65	1384.67	**0.004****
**MEHHP (ng/mL)**	58.96	50.88	39.27	44.11	53.81	0.474
**MEOHP (ng/mL)**	28.87	8.74	18.65	16.46	21.49	0.368
**MECPP (ng/mL)**	106.22	42.24	44.26	57.40	106.97	0.696
**MCMHP (ng/mL)**	29.88	8.42	18.00	62.54	19.35	0.538
**ΣDEHPm (nmole/mL)**	0.90	0.51	0.61	0.68	5.64	**0.004****
**ΣDBPm (nmole/mL)**	0.54	0.23	0.16	0.42	0.29	0.221

[a] ANCOVA adjusted for urinary TDGA, urinary creatinine, age, sex, passive smoking exposure, BMI, parental employment at petrochemical complex, and home location close to a main road.

[b] Comparison of participants at different schools using ANCOVA. *$p < 0.05$, **$p < 0.01$, ***$p < 0.001$.

Supplementary Table S3. Comparison of urinary phthalate levels for participants (N = 257) from different schools by median.

	**A, B (N=100)**	**C, D (N=97)**	**E (N=60)**	***P* value[a]**
**MMP (ng/mL)**	16.32	22.91	15.78	**<0.001*****
**MEP (ng/mL)**	13.22	11.10	10.24	0.338
**MiBP (ng/mL)**	13.75	8.60	10.10	**0.029***
**MnBP (ng/mL)**	20.16	15.13	18.17	0.086
**MEHP (ng/mL)**	10.15	14.61	208.92	**<0.001*****
**MEHHP (ng/mL)**	25.90	22.74	24.47	0.394
**MEOHP (ng/mL)**	13.81	15.30	14.18	0.517
**MECPP (ng/mL)**	28.47	25.28	80.77	**<0.001*****
**MCMHP (ng/mL)**	10.46	11.27	13.84	0.470
**ΣDEHPm (nmole/mL)**	0.36	0.38	1.22	**<0.001*****
**ΣDBPm (nmole/mL)**	0.16	0.11	0.14	**0.045***

[a] Comparison of different school groups using the Kruskal–Wallis test. *$p < 0.05$, **$p < 0.01$, ***$p < 0.001$.

Supplementary Table S4. Comparison of urinary phthalate levels for participants (N = 257) from different schools by mean after adjusting for covariance[a].

	**A, B (N=100)**	**C, D (N=97)**	**E (N=60)**	***P* value[b]**
**MMP (ng/mL)**	18.34	40.25	17.92	0.591
**MEP (ng/mL)**	54.84	92.39	39.28	0.778
**MiBP (ng/mL)**	41.66	27.60	24.46	0.108
**MnBP (ng/mL)**	50.45	31.18	39.02	0.441
**MEHP (ng/mL)**	42.86	45.14	1384.67	**0.003****
**MEHHP (ng/mL)**	55.73	41.17	53.81	0.438
**MEOHP (ng/mL)**	20.82	17.79	21.49	0.894
**MECPP (ng/mL)**	80.62	49.41	106.97	0.321
**MCMHP (ng/mL)**	21.30	35.45	19.35	0.994
**ΣDEHPm (nmole/mL)**	0.74	0.64	5.64	**0.003****
**ΣDBPm (nmole/mL)**	0.41	0.26	0.29	0.158

[a] ANCOVA adjusted for urinary TDGA, urinary creatinine, age, sex, passive smoking exposure, BMI, and parental employment at the petrochemical complex.

[b] Comparison of different school groups using ANCOVA. *$p < 0.05$, **$p < 0.01$, ***$p < 0.001$.

Supplementary Table S5. Comparison of estimated daily phthalate intake (μg/kg/day) for participants (N = 257) from different schools.

	**A, B (N=100)**	**C, D (N=97)**	**E (N=60)**	***P* value[a]**
**Median**				
**DEHP**	4.45	3.69	13.75	**<0.001**[***]
**DnBP**	0.50	0.34	0.48	**0.005**[**]
**DiBP**	0.44	0.26	0.33	**0.003**[**]
**DEP**	0.41	0.32	0.26	0.250
**DMP**	0.39	0.63	0.41	**<0.001**[***]
**Mean[b]**				
**DEHP**	6.21	6.10	80.44	**0.007**[**]
**DnBP**	0.97	0.79	0.91	0.665
**DiBP**	1.06	0.95	0.68	0.230
**DEP**	1.24	3.30	1.04	0.963
**DMP**	0.46	0.91	0.50	0.249

[a] ANCOVA for mean/Kruskal–Wallis test for median. *$p < 0.05$, **$p < 0.01$, **$p < 0.001$. [b] ANCOVA adjusted for age, sex, passive smoking exposure, BMI, and parental employment at petrochemical complex.

Supplementary Table S6. Comparison of hazard quotients and hazard index by tolerable daily intake for participants (N = 257) from different schools.

	**A, B (N=100)**	**C, D (N=97)**	**E (N=60)**	***P* value[a]**
**Median**				
**HQ_{DEHP}**	0.09	0.07	0.27	**<0.001*****
**HQ_{DnBP}**	0.05	0.03	0.05	**0.005****
**HQ_{DiBP}**	0.04	0.03	0.03	**0.003****
**HQ_{DEP}**	0.001	0.001	0.001	0.250
**HI_{hep}**	0.24	0.20	0.72	**<0.001*****
**HI_{rep}**	0.24	0.17	0.41	**<0.001*****
**Mean[b]**				
**HQ_{DEHP}**	0.12	0.12	1.61	**0.007****
**HQ_{DnBP}**	0.10	0.08	0.09	0.665
**HQ_{DiBP}**	0.11	0.10	0.07	0.230
**HQ_{DEP}**	0.002	0.007	0.002	0.963
**HI_{hep}**	0.40	0.34	4.22	**0.006****
**HI_{rep}**	0.33	0.30	1.77	**0.010****

[a] ANCOVA for mean/Kruskal–Wallis test for median. *$p < 0.05$, **$p < 0.01$, ***$p < 0.001$.

[b] ANCOVA adjusted for age, sex, passive smoking exposure, BMI, and parental employment at petrochemical complex.

Supplementary Table S7. Annual levels of air vinyl chloride monomers (VCM) and 1,1-dichloroethane at petrochemical complex and surrounding region (schools and community)[a] from May 2012 to June 2014 per local EPA of Yunlin County, Taiwan.

Location	Sampling sites [b]	VCM		1-1dichloroethane	
		Annual average (ppb)	Range (ppb)	Annual average (ppb)	Range (ppb)
Inside or close to the petrochemical complex	[1] Coast Guard North at Mai-Liao County (upwind)	3.64	ND[d]~112.83	0.5	ND [d]~6.47
	[2] Western Region, Dock (Loading products)	3.76	ND~193.6	0.77	ND~26.91
	[3] Coast Guard South at	3.46	ND~249.69	0.67	ND~10.92

	Tai-Shi County (Downwind)[1]				
Outside the petrochemical complex	[4] School A (Old campus) & School B	2.19	ND~165.82	1.05	ND~3.58
	[5] School C	1.9	ND~17.0	-	-
	[6] Division of School D	2.81	ND~76.62	0.65	ND~6.4
	[7] Community (North region)	1.77	ND~127.82	0.44	ND~3.44

[a]No ambient air VCM monitoring data was available for school E (~8.6 km) because only one sampling site, Coast Guard South, in Tai-Shi County was included in the air monitoring program by the local EPA of Yunlin County, Taiwan; [b]The exact locations of seven sampling sites are

shown in Supplementary Figure S1; [c]Only one month of data for summer (2014) was available for school C, Mai-Liao County; [d]ND: not detectable; MDL: 0.22 ppb (VCM); 0.17 ppb (1,1-dichloroethane; Huang et al., 2016).

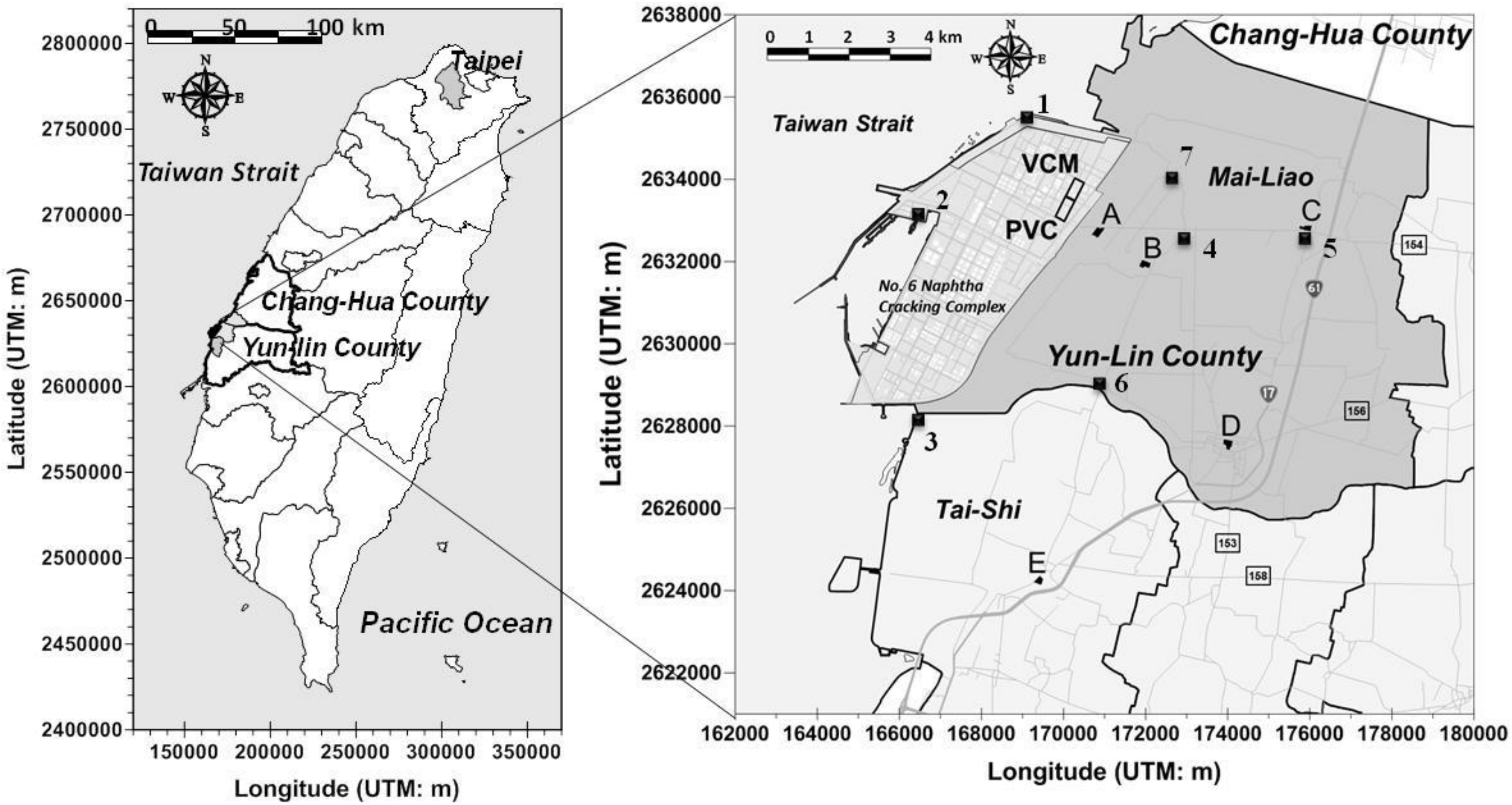

Supplementary Figure S1. Locations of air VCM and 1,1-dichloroethane monitoring sites inside (1–3) and outside (4–7) petrochemical complex (No. 6 Naphtha Cracking Complex) in central Taiwan. Air monitoring data were recorded by the local EPA of Yunlin County, Taiwan, from May 2012 to June 2014 (Huang et al., 2016).
